# Macro- and micro-geographical genetic variation in early-fitness traits in populations of maritime pine (*Pinus pinaster*)

**DOI:** 10.1093/aob/mcae190

**Published:** 2024-10-28

**Authors:** Aida Solé-Medina, Agathe Hurel, Camilla Avanzi, Santiago C González-Martinez, Giovanni G Vendramin, Francesca Bagnoli, Andrea Piotti, Maurizio Marchi, Ilaria Spanu, Juan José Robledo-Arnuncio, José Alberto Ramírez-Valiente

**Affiliations:** Institute of Forest Sciences (ICIFOR-INIA), CSIC, Madrid, Spain; Federal Research and Training Centre for Forests, Natural Hazards and Landscape, Vienna, Austria; Institute of Biosciences and BioResources, National Research Council, Sesto Fiorentino, Italy; National Research Institute for Agriculture, Food and the Environment (INRAE); University of Bordeaux; BIOGECO Cestas, France; Institute of Biosciences and BioResources, National Research Council, Sesto Fiorentino, Italy; Institute of Biosciences and BioResources, National Research Council, Sesto Fiorentino, Italy; Institute of Biosciences and BioResources, National Research Council, Sesto Fiorentino, Italy; Institute of Biosciences and BioResources, National Research Council, Sesto Fiorentino, Italy; Institute of Biosciences and BioResources, National Research Council, Sesto Fiorentino, Italy; Institute of Forest Sciences (ICIFOR-INIA), CSIC, Madrid, Spain; Institute of Forest Sciences (ICIFOR-INIA), CSIC, Madrid, Spain

**Keywords:** Adaptive divergence, climate adaptation, common garden, emergence, intraspecific genetic variation, *Pinus pinaster*, micro-geographical variation, regeneration

## Abstract

**Background and Aims:**

Assessing adaptive genetic variation and its spatial distribution is crucial to conserve forest genetic resources and manage species’ adaptive potential. Macro-environmental gradients commonly exert divergent selective pressures that enhance adaptive genetic divergence among populations. Steep micro-environmental variation might also result in adaptive divergence at finer spatial scales, even under high gene flow, but it is unclear how often this is the case. Here, we assess genetic variation in early-fitness traits among distant and nearby maritime pine (*Pinus pinaster*) populations, to investigate climatic factors associated with trait divergence, and to examine trait integration during seedling establishment.

**Methods:**

Open pollinated seeds were collected from seven population pairs across the European species distribution, with paired populations spatially close (between <1 and 21 km) but environmentally divergent. Seeds were sown in semi-natural conditions at three environmentally contrasting sites, where we monitored seedling emergence, growth and survival.

**Key Results:**

At large spatial scales, we found significant genetic divergence among populations in all studied traits, with certain traits exhibiting an association with temperature and precipitation gradients. Significant trait divergence was also detected between pairs of nearby populations. In addition, we found consistent trait correlations across experimental sites; notably, heavier seeds and earlier seedling emergence were both associated with higher seedling survival and fitness over two years in all experimental conditions.

**Conclusions:**

We identified mean annual temperature and precipitation seasonality as potential drivers of *P. pinaster* population divergence in the studied early-life traits. Populations genetically diverge also at local spatial scales, potentially suggesting that divergent natural selection can override gene flow along local-scale ecological gradients. These results suggest the species exhibits substantial adaptive potential that has allowed it to survive and evolve under contrasting environmental conditions.

## INTRODUCTION

Estimated climate change risks for forest tree species are growing across Europe, especially at lower latitudes (IPCC, 2014). Temperature rise and annual rainfall reduction, particularly during summer months, are already increasing aridification in this region (Vicente-Serrano *et al.*, 2014). Climate projections indicate that this trend will be exacerbated in the next decades (Giorgi and Lionello, 2008; Somot *et al.*, 2008), with droughts of increasing intensity, frequency and duration, coupled with a higher frequency of extreme climatic events such as heatwaves (Sheffield and Wood, 2008; Polade *et al.*, 2014). Even though tree species inhabiting drier European areas possess traits that allow them to cope with water stress (e.g. deep rooting, sclerophylly, resistance to cavitation; see Larcher, 2000; Nardini *et al.*, 2014; Moran *et al.*, 2017 and references therein), large diebacks have been documented in the region as a result of extreme drought events (Carnicer *et al.*, 2011), which may affect the long-term population persistence and alter species distributions (EEA, 2017; Sánchez-Salguero *et al.*, 2018). Consequently, a deeper knowledge of tree genetic variation in ecologically important traits and its interaction with environmental factors is becoming of primary importance for adaptive forest management, as well as for the conservation of forest genetic resources potentially valuable in current and future environments (Aitken *et al.*, 2008; Lefèvre *et al.*, 2013; Razgour *et al.*, 2019).

Differential selective pressures across species distribution ranges can lead to the evolutionary divergence of populations (Lynch and Walsh, 1998; White *et al.*, 2007), and the climate itself has been identified as a major driver of adaptive population genetic variation in forest tree species (see Alberto *et al.*, 2013; Ramírez-Valiente *et al.*, 2022 and references therein). Predictions of the impacts of future climatic conditions on forest tree populations and species will therefore benefit from a better understanding of how the historical climate has shaped standing genetic variation (Chevin *et al.*, 2010; Moran *et al.*, 2017). For this purpose, in addition to investigating macro-climatic determinants of adaptive population genetic differentiation, it is important to consider smaller spatial scales of analysis. In fact, micro-geographical adaptation along steep local environmental gradients under gene flow might be more prevalent than commonly thought and it remains understudied (Richardson *et al.*, 2014; Gauzere *et al.*, 2020; Scotti *et al.*, 2022).

Common garden and reciprocal transplant experiments are needed for disentangling the effects of genetic and environmental factors on phenotypic variation (White *et al.*, 2007; Li *et al.*, 2017) and have been widely used to assess forest trees’ climatic adaptation and potential responses to climate change (e.g. Ramirez-Valiente *et al.*, 2010; Wilczek *et al.*, 2014; Sáenz-Romero *et al.*, 2019; Fréjaville *et al.*, 2019; Patsiou *et al.*, 2020). These experiments also allow the exploration of genetic and environmental effects on trait covariation and life history strategies. In particular, when replicated across multiple sites, common garden experiments may provide insights into the environmental factors that affect trait integration and trade-offs, which are key to forecast the evolutionary potential of populations and species under variable environmental conditions (Sgrò and Hoffmann, 2004; Matesanz *et al.*, 2021). A potential caveat is that experimental conditions during plant development may impact trait expression and trait covariation (Solé-Medina *et al.*, 2022), and therefore it may be risky to extrapolate inferences under controlled conditions to natural environments, highlighting the need to establish the common gardens under conditions that mimic natural environments as much as possible.

Common garden experiments provide knowledge of tree phenotypic variation that is essential to improve predictions of species distribution models under climate change (Valladares *et al.*, 2014; Benito Garzón *et al.*, 2019; Liepe *et al.*, 2022). However, much of the available information on intraspecific genetic variation is based on adult tree traits (Gibson *et al.*, 2016; Benito Garzón *et al.*, 2019), overlooking intraspecific variation in early life stages. The transition from seed to established seedling is a critical period in the life cycle of a plant, subjected to strong selective pressures (Verdú and Traveset, 2005; Petit and Hampe, 2006). In fact, less than 10 % of viable seeds usually reach the seedling stage and survive the first year in natural conditions, or even less than 1 % in harsh environments (e.g. Castro *et al.*, 2005; Vizcaíno-Palomar *et al.*, 2014). Seedling emergence success and its timing may strongly determine survival rates at later ages (Baskin and Baskin, 1998; Donohue *et al.*, 2010) and overall recruitment success Manso *et al*., 2013*a*, *b*). Moreover, because seed-to-seedling stages are highly sensitive to environmental cues, the regeneration niche of a species is often narrower than the adult survival niche (Baskin and Baskin, 1998; Walck *et al.*, 2011; Solé-Medina *et al.*, 2020), and it may exhibit genetically determined differences among populations adapted to different environments (Baskin and Baskin, 1998; Correia *et al.*, 2014). Thus, conducting common-garden experiments to assess intraspecific variation in seedling emergence and survival during early life stages represents a necessary addition to build more comprehensive predictions of forest tree species adaptation to future climates (Climent *et al.*, 2021).

In this study, we used a multi-site common garden experiment to assess genetic variation and differentiation in early-fitness traits across maritime pine (*Pinus pinaster*) populations at large and local scales, as well as to infer potential climatic factors associated with observed trait divergence and to identify trait associations during seedling establishment. Maritime pine is a thermophilous conifer distributed in the western Mediterranean Basin and southern European Atlantic coast, from sea level up to 1800 m a.s.l., occupying a broad range of environments including oceanic, Mediterranean and semi-arid climates and a high variety of soils (Barbero *et al.*, 1998). Studies on this species have revealed substantial population genetic variation, phenotypic plasticity and genotype-by-environment interaction (i.e. differences in phenotypic plasticity among populations), in a variety of traits, particularly in adult trees (Alía *et al.*, 1995, 1997; Alía and Moro, 1996; González-Martínez *et al.*, 2002; Correia *et al.*, 2010; Zas *et al.*, 2020; Ramírez-Valiente *et al.*, 2022 and references therein). In addition, some authors have reported significant associations between population trait divergence and differences in abiotic factors such as precipitation, temperature or altitude (Alía *et al.*, 1997; Correia *et al.*, 2008; Sánchez-Salguero *et al.*, 2018), whereas others have found non-significant or weak correlations between traits and climate (Correia *et al.*, 2010; Gaspar *et al.*, 2013; Vizcaíno-Palomar *et al.*, 2016). Species distribution models based on traits measured on adult trees in common garden experiments predict a reduction of the species range in the future as a result of more adverse climatic conditions (Serra Varela, 2017). In addition, evidence of regeneration failure has been recorded in populations from Spain and the French Atlantic coast (Ribeiro *et al.*, 2022). Although the species regeneration capacity has also been the focus of several studies (Juez *et al.*, 2014; Fernandes *et al.*, 2017; Moreno-Fernández *et al.*, 2018), especially in relation to disturbances such as fire (Fernandes and Rigolot, 2007; Vega *et al.*, 2009, 2010; Maia *et al.*, 2012; Sagra *et al.*, 2018, 2019), there are comparatively fewer studies on trait population divergence at the seed-to-seedling stage in natural or semi-natural conditions (Correia *et al.*, 2014; Vizcaíno-Palomar *et al.*, 2014). All these characteristics make *P. pinaster* an interesting study system to investigate potential early fitness trait divergence associated with climatic variation. Moreover, despite evidence on range-wide genetic divergence in this wind-pollinated and wind-dispersed species, the spatial scale below which gene exchange may override divergent natural selection precluding population divergence is unclear. Recent studies based on molecular markers have suggested that selection can override gene flow in conifers, including *P. pinaster*, at local spatial scales (Scotti *et al.*, 2022; Budde *et al.*, 2023). However, whether local-scale molecular differentiation translates into divergent phenotypic trait expression and the ecological drivers involved have not been tested.

Aiming to increase the knowledge on how natural selection has shaped standing genetic variation and to identify the potential climatic factors involved in the process, we assessed quantitative trait variation among distant and nearby *P. pinaster* populations under three contrasting semi-natural common garden environments. We evaluated population differentiation in early-fitness traits spanning from seedling emergence to seedling establishment over two years. Specifically, we addressed the following questions: (1) What is the extent of intraspecific genetic variation in early-fitness traits among *P. pinaster* populations and does it correlate with provenance climate? (2) Do genetic differences in early-fitness traits exist among nearby but ecologically contrasting populations? (3) Does the growing environment alter patterns of population differentiation (i.e. is there a genotype-by-environment interaction)? (4) Do different early-fitness traits covary across populations and environments?

## MATERIALS AND METHODS

### Population sampling and common garden experiments

**Fig. 1.**
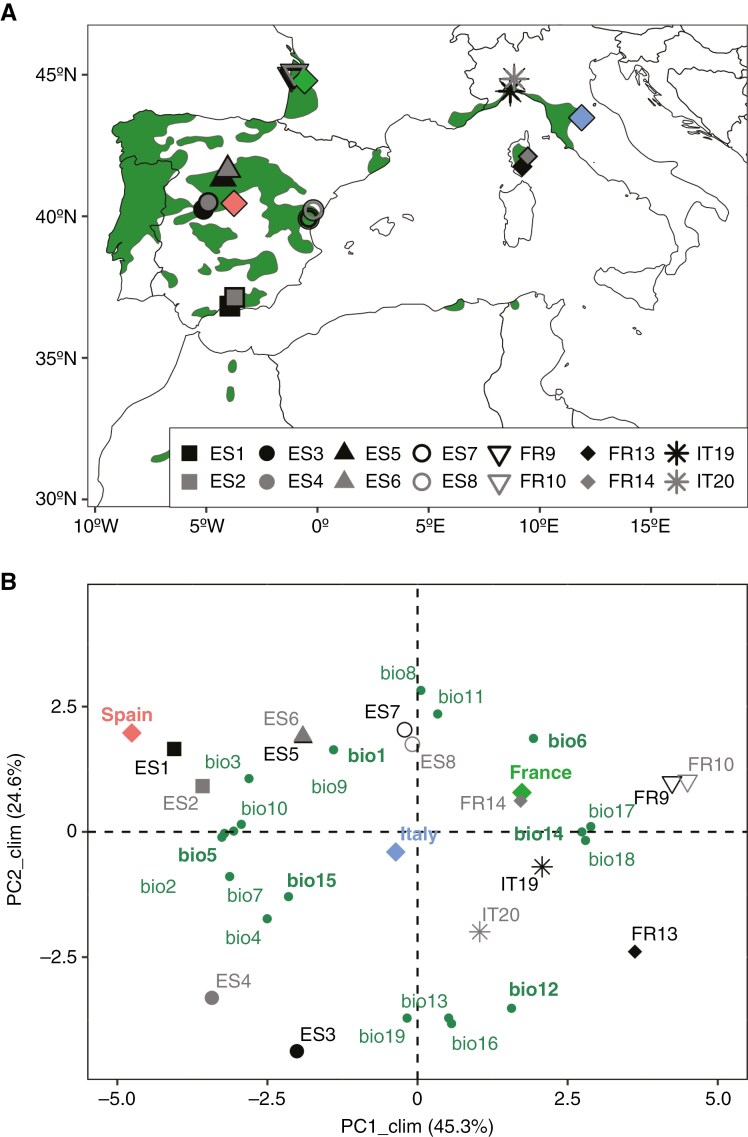
(A) Location of the 14 sampled *Pinus pinaster* populations (the two populations of each local pair shown in black and grey, sharing the same symbol) and the three common garden experiments (red, green and blue diamonds). The green area indicates the current species distribution range (EUFORGEN, 2009). (B) Principal component analysis of scaled and centred climatic variables of *P. pinaster* populations. Population scores and common garden scores are represented by the same symbols used in the top panel. Loadings for the climatic variables are in green. Selected climatic variables for correlation analysis are in bold. The climatic variables were annual mean temperature (bio1), mean diurnal range (bio2), isothermality (bio3), temperature seasonality (bio4), maximum temperature of warmest month (bio5), minimum temperature of coldest month (bio6), temperature annual range (bio7), mean temperature of wettest quarter (bio8), mean temperature of driest quarter (bio9), mean temperature of warmest quarter (bio10), mean temperature of coldest quarter (bio11), annual precipitation (bio12), precipitation of wettest month (bio13), precipitation of driest month (bio14), precipitation seasonality (bio15), precipitation of wettest quarter (bio16), precipitation of driest quarter (bio17), precipitation of warmest quarter (bio18) and precipitation of coldest quarter (bio19), extracted from CHELSA (Karger *et al.*, 2020) for the reference period 1970–2000.

In 2016, we collected seeds from 14 natural populations of *Pinus pinaster* Ait. across the European distribution of the species to establish a network of three common garden experiments (Fig. 1).

The sampling design was intended to test for trait genetic divergence among populations along both macro- and micro-environmental gradients. First, selected populations spanned most of the climatic range and the genetically distinct groups reported for the species (Jaramillo-Correa *et al.*, 2015; Serra-Varela *et al.*, 2015). Second, the design included seven pairs of populations, with paired populations being spatially close (from <1 to 21 km) but ecologically differentiated in elevation, climate and/or soil water availability (Fig. 1; Table 1). Within each population, we sampled cones from 13–25 dominant or co-dominant trees separated by at least 30 m to reduce relatedness among maternal families. In the laboratory, we extracted seeds from cones, removed the wings, and discarded empty and undeveloped seeds by water flotation. Viable seeds from each mother tree were stored separately in dry conditions at 4 °C until sowing.

**Table 1.** Geographical coordinates and main climatic features of the 14 studied *Pinus pinaster* populations. The distance and main local ecological differences between paired populations are shown.

Population	Latitude	Longitude	Distance between paired populations	Ecological contrast between paired populations	
ES1	36.827°	−3.941°	1.7 km	Altitude	Low altitude: 434 m a.s.l.
ES2	36.835°	−3.924°			High altitude: 721 m a.s.l.
ES3	40.245°	−5.122°	6.3 km	Altitude	High altitude: 1074 m a.s.l.
ES4	40.189°	−5.108°			Low altitude: 650 m a.s.l.
ES5	41.336°	−4.246°	1 km	Water availability	Dry. Dune-top position, dry sandy soil
ES6	41.341°	−4.235°			Wet. Dune-bottom position, aquifer discharge area
ES7	39.917°	−0.394°	0.7 km	Water availability	Dry. South-facing slope, aspect: 202. Intense solar radiation
ES8	39.912°	−0.389°			Wet. North-west-facing slope, aspect: 298
FR9	44.968°	−1.164°	21.5 km	Water availability	Dry. Dune-top position, dry sandy soil
FR10	44.78°	−1.23°			Wet. Dune-bottom position, aquifer discharge area
FR13	41.756°	9.212°	7.7 km	Altitude	High altitude: 924 m a.s.l.
FR14	41.816°	9.258°			Low altitude: 425 m a.s.l.
IT19	44.418°	8.671°	14.8 km	Climate	Coastal. South-facing. More rain and milder temperatures in summer and winter
IT20	44.551	8.645			Interior. North-facing. Semi-continental climate with higher diurnal and yearly temperature range

We installed the common garden experiments in three climatically contrasting sites within the distribution range of the species, in central Spain (Madrid; latitude 40.457°, longitude −3.752°), south-western France (Bordeaux; 44.786°, −0.577°) and central Italy (Arezzo; 43.481°, 11.879°) (Fig. 1; Supplementary Data Table S1). Madrid was the driest and hottest site, especially in summer months. Bordeaux presented lower temperature oscillations throughout the year and higher precipitation during summer, while Arezzo presented an intermediate climate but the highest annual precipitation (Fig. 1). The three gardens followed the same latinized row–column design with seven columns and 42 rows organized in three complete blocks. The experimental unit consisted of 16 seeds individually sown in adjacent 30-cm^2^ octagonal cells of plastic grids opened in the bottom and lateral walls (Guttagarden, Gutta, Italy). In three populations (ES5, FR9 and IT20), those geographically closest to the experimental sites, family structure was maintained in the design for purposes that go beyond the objectives of this study, with a total of 21, 24 and 20 families for ES5, FR9 and IT20, respectively, and one experimental unit per maternal family per population within each block. For each of the 11 remaining populations, we randomly sampled 144 seeds from a lot formed by pooling an equal number of seeds from every maternal family, which were then sown in three experimental units per block. In total, 4714 seeds were sown in each common garden. The experimental design was constructed using CycDesigN software (Whitaker *et al.*, 2002). The common gardens were installed in open areas with flat topography and full sun exposure, where around 5 cm of soil was removed to place the grids, subsequently filled with sieved local soil.

Before sowing, we measured the individual mass of seeds from the three populations with family structure (ES5, FR9 and IT20), as well as mean population seed mass for the remaining populations, based on the total weight of 50 randomly sampled seeds. Seeds were sown in spring 2018 in Spain and France and in spring 2019 in Italy. All experimental sites were watered for 1 month to ensure enough germination and avoid very small sample sizes, which would have precluded further analyses (see Vizcaíno-Palomar *et al.*, 2014; Solé-Medina *et al.*, 2020). All experimental sites were regularly weeded to reduce vegetative competition and were fenced to prevent herbivory.

### Measured traits

The experiment lasted 2 years from seed sowing. We monitored emergence, epicotyl elongation, dwarf shoot development (marking an important ontogenetic phase change, Climent *et al.*, 2013) and survival every 2 or 3 d from sowing until the first winter, when seedlings stopped growing. Then, we continued monitoring the same traits every 7–15 d until the end of the experiment. Height, defined as the stem length above the cotyledons (to account for differences in seed sowing depth), was measured on all alive seedlings in winter of the two studied years. Based on these measurements, we estimated the phenotypic and fitness variables described in Table 2.

**Table 2.** Early phenotypic and fitness traits in the *Pinus pinaster* common garden experiments.

Abbreviation	Measured trait	Description
Emergence	Emergence rate	Number of emerged seedlings/number of sown seeds (binomial)
Emergence_100_	Emergence rate in the first 100 d after sowing	Number of emerged seedlings in the first 100 d/number of sown seeds (binomial)
Emerg. time	Emergence time	Time from sowing to emergence (d)
Emerg. time_100_	Emergence time in the first 100 d after sowing	Time from sowing to emergence for seedlings that emerged in the first 100 d (d)
Survival_1_	Survival at the end of the first growing season (GS) (for the seedlings emerged in spring–early summer of the first GS)	Number of seedlings alive at the end of the first GS/number of seedlings that emerged in the first 100 d (binomial)
Survival_2_	Survival at the end of the second GS	Number of seedlings alive at the end of the second GS/number of seedlings alive at the end of the first GS (binomial)
Fitness_1_	Fitness at the end of the first GS (for the seedlings emerged in spring–early summer of the first GS)	Number of seedlings alive at the end of the first GS that emerged in the first 100 d/number of seeds sown (binomial)
Fitness_2_	Fitness at the end of the second GS	Number of seedlings alive at the end of the second GS/number of seeds sown (binomial)
Height_1_	Height at the end of the first GS	Height of alive seedling (above the cotyledons) at the end of the first GS (cm)
Height_2_	Height at the end of the second GS	Height of alive seedling (above the cotyledons) at the end of the second GS (cm)
Growth	Height growth	Height_2_ − Height_1_ (cm)
Develop. time	Developmental time	Time from emergence to epicotyl elongation (d)
Dwarf shoot	Dwarf shoot rate	Number of seedlings that developed dwarf shoots/number of seedlings that were alive when the first dwarf shoot appeared in the garden (binomial)

**Fig. 2.**
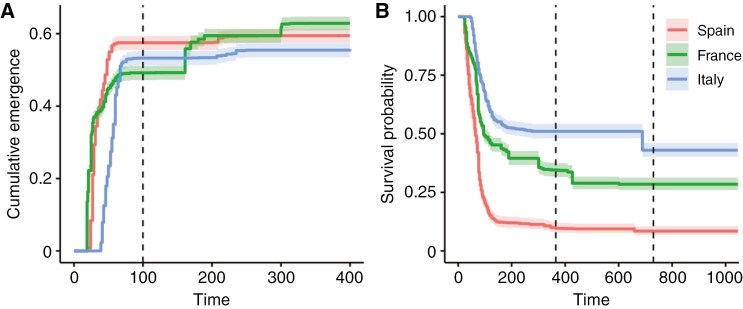
Kaplan–Meier curves for cumulative emergence (A) and survival probability of emerged *Pinus pinaster* seedlings (B) in the Spanish, French and Italian experimental sites. Time represents days since sowing. Dashed vertical lines indicate the first 100 d after sowing (A) and the end of the first and second year of the experiment (B). Shaded areas represent 95 % confidence intervals.

We considered two different reference periods for the analysis of emergence: the whole duration of the experiment and the first 100 d after sowing. Almost 90 % of overall emergence occurred within the first 100 d of the experiment, virtually stopping thereafter for 2 months, until small peaks of late emergence occurred at the end of the first growing season and even during the second year in one site (see Fig. 2). The two defined reference periods allowed clearer quantification of the fate of early-emerging seedlings (the great majority) during the first growing season (spring–early summer) and of all seedlings, including those with late emergence in the subsequent favourable periods (first-year late summer to autumn and second-year spring).

### Analysis of population differentiation and phenotypic plasticity

We tested for differences in binomial variables (emergence, survival, fitness and dwarf shoot rate) among populations and sites, and their interaction, using binomial mixed models with logit link functions (R package glmmTMB; Brooks *et al.*, 2017). We considered population, experimental site and their interaction as fixed-effect factors, and family nested within population, as well as row, column and block nested within site as random-effect factors. We implemented linear mixed models for the rest of the variables (emergence time, developmental time, growth and height), using the same factor structure described above. We considered the inverse of population sample sizes as sampling weights in the models to account for the overrepresentation of populations with family structure (Hahs-Vaughn, 2006). We also conducted Cox proportional hazard mixed-effect models (Cox, 1972) to explore temporal patterns of seedling emergence and mortality, based on the same model structure (R package coxme; Therneau, 2015). We applied Benjamini and Hochberg’s (1995) false discovery rate (FDR) correction for multiple testing as implemented in R’s built-in function *p.adjust.* When significant genetic differences among populations were found in the mixed-effect models for a given trait (i.e. when the population or the population-by-site factors were significant), we fitted new models to assess population differences within sites and extracted population means.

### Analysis of trait–climate and trait–trait associations

To explore the associations between trait genetic divergence and climatic variation among populations, we selected six climatic variables to conduct univariate analyses based on their ecological importance for seed germination and early-fitness traits in Mediterranean species (Céspedes *et al.*, 2012; Barrio-Anta *et al.*, 2020; Solé-Medina *et al.*, 2022). The six climatic variables were: annual mean temperature (bio1), maximum temperature of warmest month (bio5), minimum temperature of coldest month (bio6), annual precipitation (bio12), precipitation of driest month (bio14) and precipitation seasonality (bio15). We performed Pearson correlations among population trait means and individual climatic variables and implemented FDR correction for multiple testing.

Finally, we explored trait–trait associations within sites using Pearson correlations on populations’ mean trait values, conducted FDR correction and plotted them with network graphs (R package igraph; Csardi and Nepusz, 2006).

### Analysis of population differentiation at local scale

We examined trait divergence at local spatial scale between paired populations with the same rationale explained for all populations, but with a modified model structure. For this analysis, we considered region (categorical factor grouping paired populations), experimental site and their interaction as fixed-effect factors, and population nested within region, family nested within population, as well as row, column and block nested within site as random-effect factors. When the inclusion of the random factor *population nested within region* significantly improved the models after FDR correction, we re-ran the models including it as a fixed-effect factor and extracted population means. The same procedure was implemented within experimental sites. We then ran post-hoc Tukey tests (R package multcomp, Hothorn *et al.*, 2008), to test for differences among paired populations.

## RESULTS

### Trait variation across experimental sites

After FDR correction, mixed models revealed significant phenotypic plasticity (i.e. a significant site factor) for all the considered seedling traits except second-year survival, as well as significant among-population genetic variation in phenotypic plasticity (i.e. significant site × population factor) for all study traits except first-year survival (Table 3). Most seedling emergence (89.2 %) occurred within the first 100 d after sowing in all three sites (in spring and early summer), but there was a second emergence peak at the end of summer and the beginning of autumn (between 161 and 267 d after sowing), as well as a third peak in spring of the second year, especially in France (Fig. 2). Emergence occurring after the first spring accounted for around 2 % of total emergence in the Spanish and Italian sites, versus 14 % in the French site. The emergence rate was higher at the Spanish site during the first spring, becoming larger at the French site when considering the entire experimental period due to higher delayed emergence (Fig. 2).

**Table 3.** Mixed models for *Pinus pinaster* early phenotypic and fitness traits. χ^2^ and *P*-values are shown for the fixed-effect factors Site (*df* = 2), Population (*df* = 13) and Site × Population (*df* = 26). Statistically significant results after FDR correction are shown in bold. See Table 2 for trait descriptions.

	Site		Population		Site × Population	
	χ^2^	*P*	χ^2^	*P*	χ^2^	*P*
Emergence	16.5	**<0.001**	86.9	**<0.001**	147	**<0.001**
Emergence_100_	18.6	**<0.001**	124	**<0.001**	193	**<0.001**
Emerg. time	35.2	**<0.001**	11.3	0.601	854	**<0.001**
Emerg. time_100_	886	**<0.001**	41.2	**<0.001**	224	**<0.001**
Survival_1_	19.3	**<0.001**	44	**<0.001**	29.8	0.301
Survival_2_	1.82	0.424	25.4	**0.023**	49	**0.005**
Fitness_1_	23.4	**<0.001**	51.5	**<0.001**	75.9	**<0.001**
Fitness_2_	15.5	**<0.001**	52	**<0.001**	57.2	**<0.001**
Height_1_	689	**<0.001**	59.6	**<0.001**	220	**<0.001**
Height_2_	997	**<0.001**	81.4	**<0.001**	323	**<0.001**
Growth	668	**<0.001**	52.3	**<0.001**	220	**<0.001**
Develop. time	51.9	**<0.001**	4.28	0.988	360	**<0.001**
Dwarf shoot	6.84	**0.036**	32.5	**0.002**	70.2	**<0.001**

Seedling survival showed marked differences across sites (Table 3; Fig. 2). Survival at the end of the first growing season was by far the lowest in Spain, with only 12 % of the emerged seedlings alive compared to 40 and 53 % in France and Italy, respectively (Fig. 2). The highest mortality rate over the study period occurred at the beginning of the first growing season, with three-quarters of the recorded deaths occurring within the first 127 d after sowing (i.e. in late spring and summer months) (Fig. 2). In the second growing season, survival was around 75 % in all sites with no significant differences across sites (Table 3). Overall, fitness, i.e. the proportion of seedlings alive relative to the number of seeds sown, was significantly lower in Spain than in Italy and France at the end of the first and second growing seasons (Table 3). Specifically, fitness after 2 years was 5.8, 21.8 and 24.1 %, in the Spanish, French and Italian sites, respectively.

Height also showed significant differences across sites at the end of the first growing season (mean ± s.e. 4.3 ± 0.4, 2.1 ± 0.4 and 0.8 ± 0.4 cm for Italian, Spanish and French sites, respectively). At the end of the second growing season seedlings were significantly taller in the Italian site (25.4 ± 0.8 cm), while no significant differences were observed between the Spanish and French sites (10.1 ± 0.9 and 9.0 ± 0.8 cm, respectively).

### Trait variation among populations and its association with macro-climatic provenance variation

**Fig. 3.**
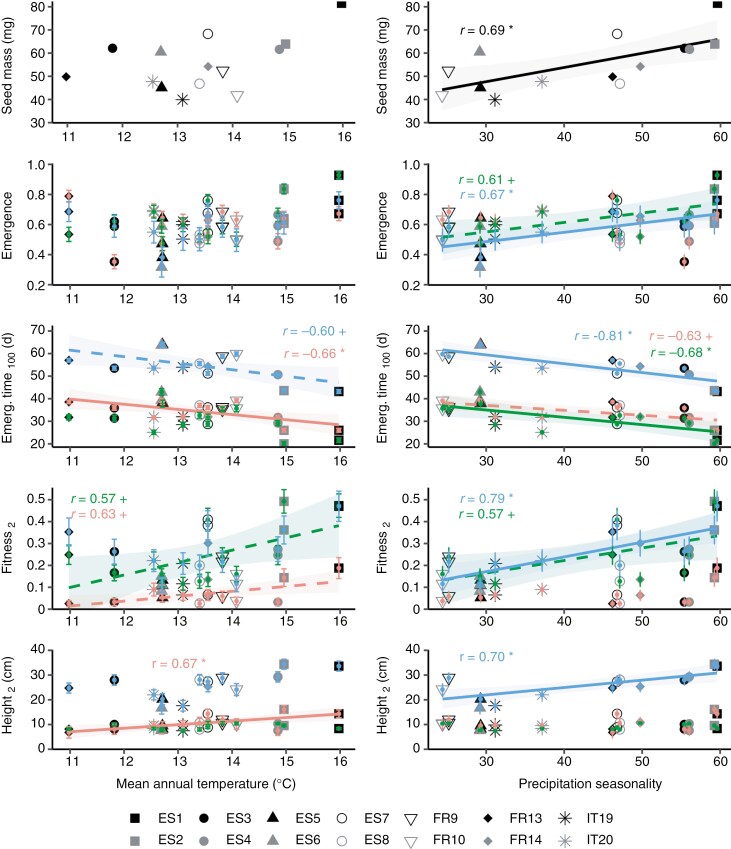
Correlations between selected climatic variables and *Pinus pinaster* seedling phenotypic traits (see Table 2 for definitions) in the Spanish, French and Italian experimental sites (red, green and blue, respectively). Significant Pearson correlation coefficients (*r*) after FDR correction are shown, with *P* values represented by ^+^*P* < 0.07, *P < 0.05, **P < 0.01 and ***P < 0.001. Shaded areas represent 95 % confidence intervals.

Most phenotypic and fitness traits showed strong genetic variation among populations (i.e. significant population effect; Table 3), with some of them showing significant association with climatic variation, particularly with mean annual temperature and precipitation seasonality (Fig. 3; Supplementary Data Fig. S1). After FDR correction, Pearson correlations showed that seedlings from provenances with higher mean annual temperature and higher precipitation seasonality tended to have higher emergence rate, shorter emergence time and higher fitness and grew taller throughout the entire experiment (Fig. 3; Fig. S1). In addition, we found a significant positive association between seed mass and precipitation seasonality (Fig. 3).

### Trait variation between local populations within regions

**Fig. 4.**
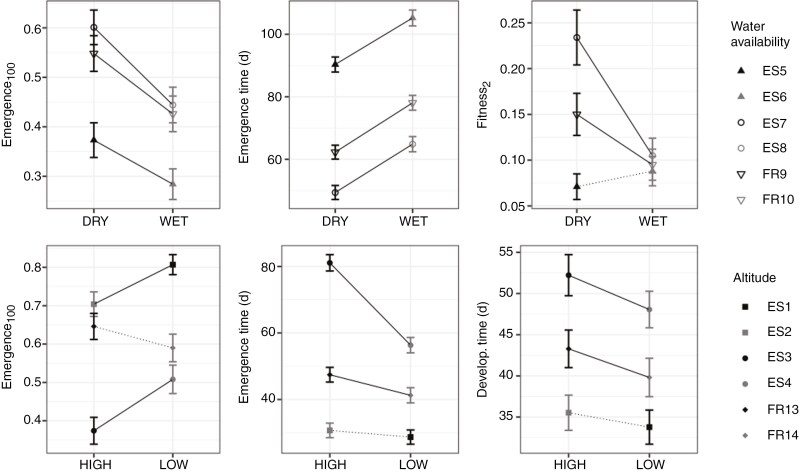
Early-life trait differentiation between spatially close but ecologically contrasting *Pinus pinaster* population pairs. The two populations of each pair are shown in black and grey, sharing the same symbol. The contrasting ecological factors were water availability (upper panels) or altitude (lower panels). Observed population means ± s.e. are shown. Solid and dashed lines indicate significant and non-significant differences after FDR correction among local population pairs, respectively.

Despite their geographical proximity within their respective regions, paired populations also exhibited significant trait divergence between each other (Supplementary Data Table S2). In particular, post-hoc Tukey tests revealed that all traits except first-year survival and dwarf shoot rate exhibited significant divergence between local populations (Table S2). Trait differences between local populations within regions were highly pair-, site- and trait-dependent (Table S2), but there were some remarkable patterns. First, in pairs where populations differed in soil water availability, the drier population of the pair showed higher emergence rate and earlier emergence, and in two out of three population pairs, the drier population also showed higher fitness at the end of the experiment (Fig. 4). This held true even for populations separated by only a few hundred meters. Second, in pairs where populations differed in elevation, the lower population tended to emerge in higher proportion and earlier and to grow taller and faster (Fig. 4).

### Trait coordination within experimental sites

**Fig. 5.**
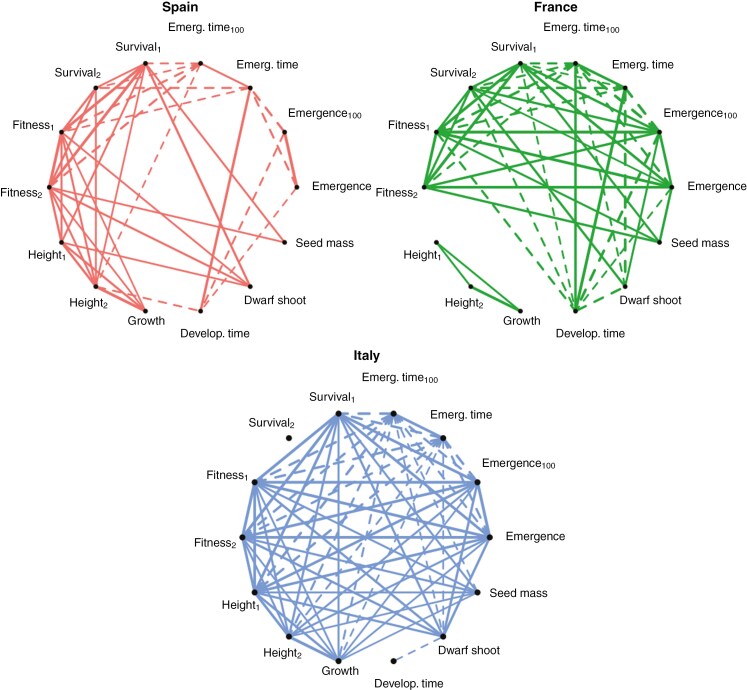
Network graphs showing statistically significant (*P* < 0.05) *Pinus pinaster* seedling trait–trait correlations in the Spanish (red), French (green) and Italian (blue) experimental sites after FDR correction. Line thickness represents the strength of the correlation, as measured by Pearson correlation coefficient. Solid and dashed lines correspond to positive and negative correlations, respectively.

Significant trait–trait correlations were common, showing strong phenotypic integration especially in the Italian and French sites (Fig. 5). Remarkably consistent patterns across sites were found regarding seed mass and emergence time (Fig. 5). Specifically, populations with heavier seeds had higher first-year survival and higher fitness at the end of the experiment in all sites. In addition, populations that emerged earlier in spring showed higher first-year survival and higher fitness in both growing seasons in all sites (Fig. 5). Fitness at the end of the first growing season was highly and positively correlated with first-year survival in all sites, and with emergence rate in the French and Italian sites (*r* = 0.94, *P* < 0.001 and *r* = 0.96, *P* < 0.001, respectively). Finally, height, proportion of dwarf shoots, survival and fitness were strongly correlated to one another in Italy and Spain. On average, populations that grew more had a higher proportion of seedlings developing dwarf shoots and higher survival and fitness rates (Fig. 5).

## DISCUSSION

Our study revealed that *P. pinaster* populations genetically differed in virtually all studied early-fitness traits, presumably because of divergent selective pressures associated with heterogeneous climatic conditions at different spatial scales across the species range. We found clinal seedling trait variation associated with annual mean temperature and precipitation seasonality of the population of origin. Overall, populations from warmer provenances with higher precipitation seasonality tended to emerge earlier, to grow taller and to show higher fitness over the two study years. We also found evidence of significant micro-geographical trait variation among populations located a few hundred metres apart along steep environmental gradients. Finally, different populations showed contrasting patterns of phenotypic plasticity for most seedling traits, but certain trait–trait correlations were largely consistent across experimental environments.

### Clinal variation of seedling traits along macroclimatic gradients

It is well established that maritime pine presents high levels of population differentiation in quantitative traits (Alía *et al.*, 1995, 1997; Chambel *et al.*, 2007; Aranda *et al.*, 2010; de Miguel *et al.*, 2022; Ramírez-Valiente *et al*., 2022 and references therein). However, few studies have focused on early-fitness traits or addressed the potential environmental drivers of population genetic divergence at different spatial scales.

Our results revealed associations between among-population variation in early-fitness traits and the climate of the populations of origin, especially with annual temperature and precipitation seasonality. Temperature and precipitation are critical environmental factors for successful recruitment, and they have previously been identified as important drivers of genetic divergence in early-fitness traits for many species (Baskin and Baskin, 1998), including pines (Ramírez-Valiente *et al.*, 2021). Beyond annual precipitation accumulation, its seasonal distribution (and its effect on soil moisture) is considered an important selective agent for germination strategies (Cowling *et al.*, 2005; Urbieta *et al.*, 2008; de Dios Miranda *et al.*, 2009). In particular, studies on *P. pinaster* natural regeneration have found precipitation timing to be a major determinant of seed germination, early seedling development and survival (Ruano *et al.*, 2009; Rodriguez-Garcia *et al.*, 2011). Our results showed that *P. pinaster* populations that have evolved under stronger seasonal precipitation regimes (characterized also by lower summer precipitation) and warmer climates had earlier seedling emergence than populations from provenances with lower annual temperature and a more homogeneous seasonal distribution of rainfall (and higher summer rainfall).

Importantly, the fact that maritime pine seedlings from warmer provenances tend to have higher fitness and grow taller at the warmest experimental site (Spain), relative to seedlings from colder provenances, suggested that they are better adapted to higher temperatures and drier conditions during early recruitment stages. In the Italian site, populations with higher precipitation seasonality showed higher and earlier emergence, higher fitness and were taller after 2 years, while in the French site we observed mixed trends with both environmental variables. These results suggest higher survival of seedlings from harsher (warmer and high seasonality in precipitation) environments irrespective of the growing site, and are consistent with those found by Alía *et al.* (1997) and Ramírez-Valiente *et al.* (2022) for adult trees. However, previous results had reported higher growth for maritime pines from (more humid) Atlantic provenances when grown in milder environments (Kremer and Roussel, 1986; Alía *et al.*, 1995, 1997; Archambeau *et al.*, 2022), including during early life stages (Fernández *et al.*, 1999). The fact that our experiments were conducted in the field under semi-natural conditions, in contrast to the chamber experiments by Fernández *et al.* (1999), illustrates the risks of generalizing results obtained under controlled conditions (see also Alía *et al.*, 2014), as well as the potentially strong dependency of field experiments on the environmental conditions experienced during the study years.

The observed patterns of covariation between seedling fitness traits and climate of the population of origin are consistent with climate-driven divergent selection across the species distribution (Davis *et al.*, 2005; Alberto *et al.*, 2013), although the influence of epigenetic or environmental maternal effects cannot be fully discarded. Seed provisioning is a well-known mechanism of transmission of environmental maternal effects in many plant species, including maritime pine (Cendán *et al.*, 2013; Zas *et al.*, 2013; Suárez-Vidal *et al.*, 2017), and we found that seed mass, which was positively correlated with seedling fitness (discussed below), was higher for seeds collected from populations with more seasonal precipitation regimes (Fig. 3). Therefore, the higher performance observed for the latter populations might have been partly mediated by differences in the maternal environment experienced by collected seeds. In any case, mean seed mass has been found to be a genetically variable and highly heritable trait in *P. pinaster*, with a comparatively weaker influence of non-genetic effects (Zas and Sampedro, 2015), suggesting that adaptive genetic factors have probably influenced the observed trends in the results. Future studies should consider investigating the relative importance of genetic versus environmental effects in seed mass variation among maritime pine populations.

### Seedling trait variation at local spatial scale

Unlike the frequent evidence of quantitative genetic divergence among distant *P. pinaster* populations, little is known on the minimum scale over which genetic divergence occurs in this (Archambeau *et al.*, 2022) or many other species (Richardson *et al.*, 2014). Our sampling design allowed us to investigate trait genetic variation at local spatial scales (from <1 km up to 21 km), a range of distances rarely considered in evolutionary ecology research because it is commonly assumed that high gene flow invariably overcomes selection at fine spatial scale (Richardson *et al.*, 2014). However, quantitative differentiation at local scale could be of the same order of magnitude as that detected in range-wide studies (Eckert *et al*., 2015).

Our results revealed genetic variation among populations located spatially close but in sharply contrasting environmental conditions. The differences found depended on the experimental site and phenotypic trait, but all paired (nearby) populations showed a certain level of significant trait divergence (Fig. 4). At this local spatial scale, with presumably high levels of gene exchange via pollen and seed dispersal (Salvador *et al.*, 2000), it is unlikely that the observed variation is explained by neutral genetic drift, but rather by locally divergent selection overcoming the homogenizing effect of gene flow (Eveno *et al.*, 2008). This was tested by Eckert *et al.* (2015), who provided strong evidence of divergent selection as the main driver of genetic differentiation among nearby populations of *Pinus lambertiana*.

Our results complement recent findings of molecular genetic differentiation at local scales in *P. pinaster* (Scotti *et al.*, 2022; Budde *et al.*, 2023), showing that genetic differentiation also occurs at the phenotypic level. Importantly, our findings suggest that selection operating at micro-geographical scales may promote genetic differentiation among highly connected populations, maintaining and enhancing the adaptive potential of the species across heterogeneous landscapes, crucial for their persistence under changing climatic conditions.

### Early fitness effects of seed mass and emergence time

Our results revealed a consistent positive association between seed mass and seedling fitness over 2 years in all studied sites (Fig. 5). Positive associations between seed mass and survival seem to be common in species inhabiting highly seasonal climates (e.g. Gómez, 2004; Moles and Westoby, 2004; Ramírez-Valiente *et al.*, 2009; Larson *et al*., 2014; Lebrija-Trejos *et al.*, 2016), including pine species (Parker *et al.*, 2006; Cendán *et al.*, 2013; Zas *et al.*, 2013). A number of studies in different species have found that larger seeds have more reserves and produce seedlings with larger growth and/or deeper roots (Surles *et al.*, 1993; Westoby *et al.*, 1996; Wennström *et al.*, 2002; Bladé and Vallejo, 2008; Leishman *et al.*, 2009; Wahid and Bounoua, 2013). Moreover, in pine species, higher seed mass has been associated with higher root investment (Matías *et al.*, 2014; Ramírez-Valiente and Robledo-Arnuncio, 2015), which is of primary importance to access deeper water sources and increase the probabilities of seedling establishment under drought conditions.

We also found evidence of higher survival and fitness of seedlings with earlier emergence, consistent with results obtained for other species inhabiting seasonal climates (Seiwa, 2000; Simons and Johnston, 2000; Donohue, 2002; Castro, 2006; Warwell and Shaw, 2019). Selection for early emergence in the growing season is expected if it provides advantages with respect to a predictable environmental cue (Verdú and Traveset, 2005; Donohue *et al.*, 2010). Early emergence is considered of great importance in Mediterranean and arid ecosystems, because it provides seedlings with more time to grow and develop before the onset of the recurrent and critical summer drought (Vizcaíno-Palomar *et al.*, 2014; Callejas-Díaz *et al.*, 2022). In fact, in our study we found a clear and consistent relationship between early emergence time in spring and seedling fitness and survival, suggesting that early emergence had an adaptive role in response to summer drought across sites, irrespectively of their climate. Importantly, both macroclimatic clines and local-scale variation among populations that differ in water availability point to the same conclusion. More arid populations (warmer and with more seasonal precipitation) emerged earlier and, at a local scale, the drier population of the pair tended to show earlier emergence across sites (although differences were not always significant), suggesting that earlier emergence is favoured under water limitation at both spatial scales.

## CONCLUSIONS

Altogether, our results showed that *P. pinaster* populations genetically differed in early life stages, with mean annual temperature and precipitation seasonality as potential macroclimatic drivers of population divergence. Evidence of micro-geographical genetic divergence among nearby populations suggests that natural selection can override gene flow under strongly contrasting environments, generating adaptive genetic variation locally within regions. Finally, consistent patterns of trait correlation across experimental sites highlight that heavier seeds and early seedling emergence elicit higher survival rates and fitness in this species. Strong genetic variation and differences in plastic responses among populations suggest high adaptive potential in *P. pinaster* at early-life stages, provided that the increasing aridification predicted in southern European regions does not critically hinder seedling survival.

## SUPPLEMENTARY DATA

Supplementary data are available at *Annals of Botany* online and consist of the following.

Table S1. Geographical coordinates and main climatic features of the 14 *Pinus pinaster* populations and three common-garden sites under study. Table S2. Population mean and standard deviation of *Pinus pinaster* seedling traits that showed significant divergence between paired nearby populations. Figure S1. Correlations between selected climatic variables and *Pinus pinaster* seedling phenotypic traits in the experimental sites.

mcae190_suppl_Supplementary_Tables_S1-S2_Figure_S1
