# Supplementary material for: Macro- and micro-geographical genetic variation in early-fitness traits in populations of maritime pine (Pinus pinaster)
Source: Ann Bot. 2024 Oct 28;135(4):655–68. doi: 10.1093/aob/mcae190 (PMC11904904; doi:10.1093/aob/mcae190)

**Supplementary Information**

**Table S1.** Geographical coordinates and main climatic features of the fourteen *Pinus pinaster* populations and three common-garden sites under study. AMT, annual mean temperature. AP, annual precipitation.

| Population and Site | Latitude (ºN) | Longitude (ºE) | Altitude (m a.s.l) | AMT (ºC) | AP (mm) |
| --- | --- | --- | --- | --- | --- |
| ES1 | 36.827 | -3.941 | 434 | 15.98 | 512 |
| ES2 | 36.835 | -3.924 | 721 | 14.96 | 545 |
| ES3 | 40.245 | -5.122 | 1074 | 11.82 | 1025 |
| ES4 | 40.189 | -5.108 | 650 | 14.85 | 1066 |
| ES5 | 41.336 | -4.246 | 825 | 12.71 | 497 |
| ES6 | 41.341 | -4.235 | 818 | 12.70 | 490 |
| ES7 | 39.917 | -0.394 | 724 | 13.55 | 527 |
| ES8 | 39.912 | -0.389 | 725 | 13.40 | 556 |
| FR9 | 44.968 | -1.164 | 40 | 13.82 | 890 |
| FR10 | 44.780 | -1.230 | 5 | 14.08 | 920 |
| FR13 | 41.756 | 9.212 | 924 | 10.98 | 1031 |
| FR14 | 41.816 | 9.258 | 425 | 13.55 | 720 |
| IT19 | 44.418 | 8.671 | 393 | 13.09 | 964 |
| IT20 | 44.551 | 8.645 | 443 | 12.55 | 953 |
| Spain (ES) | 40.457 | -3.752 | 645 | 15.3 | 426 |
| France (FR) | 44.786 | -0.577 | 15 | 14.9 | 922 |
| Italy (IT) | 43.481 | 11.879 | 268 | 14.6 | 1035 |

**Table S2.** Population mean and standard deviation (between brackets) of *Pinus pinaster* seedling traits that showed significant divergence between paired nearby populations after FDR correction, based on Tukey tests. Results are shown for each trait and population pair when conducting the analysis on all experimental sites (Total) or on each experimental site (Spain, France and Italy). (ns: non-significant divergence).

|  |  | Elevation | | Elevation | | Water availability | | Water availability | | Water availability | | Elevation | | Climate | |
| --- | --- | --- | --- | --- | --- | --- | --- | --- | --- | --- | --- | --- | --- | --- | --- |
|  |  | **ES1/ES2** | | **ES3/ES4** | | **ES5/ES6** | | **ES7/ES8** | | **FR9/FR10** | | **FR13/FR14** | | **IT19/IT20** | |
| *Emergence* | | |  |  |  |  |  |  |  |  |  |  |  |  |  |
|  | Total | 0.80 (0.02) | 0.71 (0.03) | 0.51 (0.03) | 0.59 (0.03) | ns | ns | 0.66 (0.03) | 0.52 (0.03) | 0.62 (0.03) | 0.53 (0.03) | 0.67 (0.03) | 0.60 (0.03) | 0.57 (0.03) | 0.64 (0.03) |
|  | Spain | ns | ns | ns | ns | ns | ns | ns | ns | ns | ns | ns | ns | ns | ns |
|  | France | ns | ns | ns | ns | ns | ns | ns | ns | ns | ns | ns | ns | ns | ns |
|  | Italy | ns | ns | ns | ns | ns | ns | ns | ns | ns | ns | ns | ns | ns | ns |
| *Emergence_100_* | | |  |  |  |  |  |  |  |  |  |  |  |  |  |
|  | Total | 0.81 (0.03) | 0.70 (0.03) | 0.37 (0.04) | 0.51 (0.04) | 0.37 (0.04) | 0.28 (0.03) | 0.60 (0.04) | 0.44 (0.04) | 0.55 (0.04) | 0.43 (0.04) | ns | ns | ns | ns |
|  | Spain | ns | ns | ns | ns | ns | ns | ns | ns | ns | ns | ns | ns | ns | ns |
|  | France | ns | ns | ns | ns | ns | ns | ns | ns | ns | ns | ns | ns | ns | ns |
|  | Italy | ns | ns | ns | ns | ns | ns | ns | ns | ns | ns | ns | ns | ns | ns |
| *Emergence time* | | |  |  |  |  |  |  |  |  |  |  |  |  |  |
|  | Total | ns | ns | 81.09 (2.45) | 56.33 (2.31) | 90.34 (2.40) | 105.22 (2.54) | 49.40 (2.24) | 64.85 (2.44) | 62.30 (2.21) | 78.08 (2.38) | 47.44 (2.22) | 41.25 (2.29) | ns | ns |
|  | Spain | ns | ns | 66.59 (3.14) | 44.33 (2.77) | 40.52 (2.24) | 70.98 (2.86) | 35.85 (2.74) | 55.40 (2.89) | 39.99 (2.16) | 46.61 (2.65) | 38.17 (2.39) | 30.02 (2.58) | ns | ns |
|  | France | ns | ns | 105.69 (6.05) | 71.70 (5.90) | ns | ns | 59.06 (5.66) | 74.94 (6.42) | 82.01 (5.37) | 115.20 (6.49) | ns | ns | ns | ns |
|  | Italy | ns | ns | 69.21 (2.89) | 52.77 (2.81) | ns | ns | ns | ns | 65.32 (2.44) | 76.10 (3.08) | ns | ns | ns | ns |
| *Emergence time_100_* | | | |  |  |  |  |  |  |  |  |  |  |  |  |
|  | Total | ns | ns | 40.48 (0.61) | 37.25 (0.55) | 47.15 (0.61) | 48.69 (0.65) | 38.91 (0.53) | 40.88 (0.58) | 43.21 (0.53) | 45.21 (0.58) | 42.65 (0.52) | 38.95 (0.53) | 38.22 (0.54) | 36.73 (0.51) |
|  | Spain | ns | ns | 35.92 (1.01) | 31.70 (0.89) | ns | ns | ns | ns | 36.27 (0.75) | 39.10 (0.86) | 38.55 (0.79) | 31.87 (0.83) | ns | ns |
|  | France | ns | ns | ns | ns | ns | ns | ns | ns | ns | ns | ns | ns | ns | ns |
|  | Italy | ns | ns | 53.51 (0.89) | 50.65 (0.86) | ns | ns | 51.18 (0.84) | 55.57 (0.91) | ns | ns | 57.03 (0.84) | 54.33 (0.85) | ns | ns |
| *Survival_1_* | | |  |  |  |  |  |  |  |  |  |  |  |  |  |
|  | Total | ns | ns | ns | ns | ns | ns | ns | ns | ns | ns | ns | ns | ns | ns |
|  | Spain | ns | ns | ns | ns | ns | ns | ns | ns | ns | ns | ns | ns | ns | ns |
|  | France | ns | ns | ns | ns | ns | ns | ns | ns | ns | ns | ns | ns | ns | ns |
|  | Italy | ns | ns | ns | ns | ns | ns | ns | ns | ns | ns | ns | ns | ns | ns |
| *Survival_2_* | | |  |  |  |  |  |  |  |  |  |  |  |  |  |
|  | Total | ns | ns | 0.57 (0.06) | 0.74 (0.05) | ns | ns | 0.89 (0.03) | 0.64 (0.07) | 0.79 (0.05) | 0.57 (0.07) | ns | ns | ns | ns |
|  | Spain | ns | ns | ns | ns | ns | ns | ns | ns | ns | ns | ns | ns | ns | ns |
|  | France | ns | ns | 0.51 (0.08) | 0.74 (0.07) | ns | ns | 0.49 (0.09) | 0.91 (0.04) | 0.59 (0.08) | 0.80 (0.06) | ns | ns | ns | ns |
|  | Italy | ns | ns | ns | ns | ns | ns | ns | ns | ns | ns | ns | ns | ns | ns |

Table S2 (continued)

|  |  | **ES1/ES2** | | **ES3/ES4** | | **ES5/ES6** | | **ES7/ES8** | | **FR9/FR10** | | **FR13/FR14** | | **IT19/IT20** | |
| --- | --- | --- | --- | --- | --- | --- | --- | --- | --- | --- | --- | --- | --- | --- | --- |
| *Fitness_1_* | |  |  |  |  |  |  |  |  |  |  |  |  |  |  |
|  | Total | 0.45 (0.04) | 0.33 (0.03) | ns | ns | ns | ns | 0.23 (0.03) | 0.11 (0.02) | 0.15 (0.02) | 0.10 (0.02) | ns | ns | ns | ns |
|  | Spain | ns | ns | ns | ns | ns | ns | ns | ns | ns | ns | ns | ns | ns | ns |
|  | France | ns | ns | ns | ns | ns | ns | ns | ns | ns | ns | ns | ns | ns | ns |
|  | Italy | ns | ns | ns | ns | ns | ns | ns | ns | ns | ns | ns | ns | ns | ns |
| *Fitness_2_* | |  |  |  |  |  |  |  |  |  |  |  |  |  |  |
|  | Total | ns | ns | ns | ns | ns | ns | 0.24 (0.03) | 0.10 (0.02) | 0.16 (0.02) | 0.09 (0.02) | ns | ns | ns | ns |
|  | Spain | ns | ns | ns | ns | ns | ns | ns | ns | ns | ns | ns | ns | ns | ns |
|  | France | ns | ns | ns | ns | ns | ns | 0.41 (0.05) | 0.13 (0.03) | ns | ns | 0.25 (0.05) | 0.14 (0.03) | 0.12 (0.03) | 0.22 (0.04) |
|  | Italy | ns | ns | ns | ns | ns | ns | ns | ns | ns | ns | ns | ns | ns | ns |
| *Height_1_* | |  |  |  |  |  |  |  |  |  |  |  |  |  |  |
|  | Total | ns | ns | ns | ns | ns | ns | 2.64 (0.42) | 1.31 (0.45) | 2.85 (0.42) | 2.23 (0.44) | ns | ns | 1.59 (0.42) | 2.22 (0.41) |
|  | Spain | ns | ns | ns | ns | ns | ns | ns | ns | ns | ns | ns | ns | ns | ns |
|  | France | ns | ns | ns | ns | ns | ns | ns | ns | ns | ns | ns | ns | ns | ns |
|  | Italy | 7.14 (0.79) | 6.26 (0.80) | 4.14 (0.81) | 5.27 (0.81) | ns | ns | ns | ns | 4.82 (0.80) | 3.00 (0.84) | ns | ns | ns | ns |
| *Height_2_* | |  |  |  |  |  |  |  |  |  |  |  |  |  |  |
|  | Total | ns | ns | ns | ns | ns | ns | 16.24 (0.92) | 14.20 (1.05) | ns | ns | 13.40 (0.98) | 14.96 (0.96) | 10.41 (0.97) | 13.44 (0.90) |
|  | Spain | ns | ns | ns | ns | ns | ns | ns | ns | ns | ns | ns | ns | ns | ns |
|  | France | ns | ns | ns | ns | ns | ns | ns | ns | ns | ns | ns | ns | ns | ns |
|  | Italy | ns | ns | ns | ns | ns | ns | ns | ns | ns | ns | ns | ns | ns | ns |
| *Growth* | |  |  |  |  |  |  |  |  |  |  |  |  |  |  |
|  | Total | ns | ns | ns | ns | ns | ns | ns | ns | ns | ns | ns | ns | ns | ns |
|  | Spain | ns | ns | ns | ns | ns | ns | ns | ns | ns | ns | ns | ns | ns | ns |
|  | France | 7.21 (0.37) | 8.70 (0.36) | ns | ns | ns | ns | ns | ns | ns | ns | 7.44 (0.49) | 9.98 (0.59) | ns | ns |
|  | Italy | ns | ns | ns | ns | ns | ns | ns | ns | ns | ns | ns | ns | ns | ns |
| *Develop. Time* | | |  |  |  |  |  |  |  |  |  |  |  |  |  |
|  | Total | ns | ns | ns | ns | ns | ns | ns | ns | ns | ns | ns | ns | ns | ns |
|  | Spain | ns | ns | 40.58 (2.42) | 33.14 (1.92) | 29.45 (1.82) | 44.27 (2.27) | ns | ns | 28.91 (1.50) | 34.02 (2.20) | ns | ns | ns | ns |
|  | France | ns | ns | ns | ns | ns | ns | ns | ns | ns | ns | ns | ns | ns | ns |
|  | Italy | ns | ns | ns | ns | ns | ns | ns | ns | ns | ns | ns | ns | ns | ns |
| *Dwarf shoot* | | |  |  |  |  |  |  |  |  |  |  |  |  |  |
|  | Total | ns | ns | ns | ns | ns | ns | ns | ns | ns | ns | ns | ns | ns | ns |
|  | Spain | ns | ns | ns | ns | ns | ns | ns | ns | ns | ns | ns | ns | ns | ns |
|  | France | ns | ns | ns | ns | ns | ns | ns | ns | ns | ns | ns | ns | ns | ns |
|  | Italy | ns | ns | ns | ns | ns | ns | ns | ns | ns | ns | ns | ns | ns | ns |

**Figure S1.** Correlations between selected climatic variables and *Pinus pinaster* seedling phenotypic traits in the Spanish, French and Italian experimental sites (red, green and blue, respectively). Significant Pearson correlation coefficients (*r*) after FDR correction are shown, with *P* values represented by ^+^ (*P*< 0.07)*,* * (*P*< 0.05), ** (*P*< 0.01), and *** (*P*< 0.001). Shaded areas represent 95% confidence intervals.


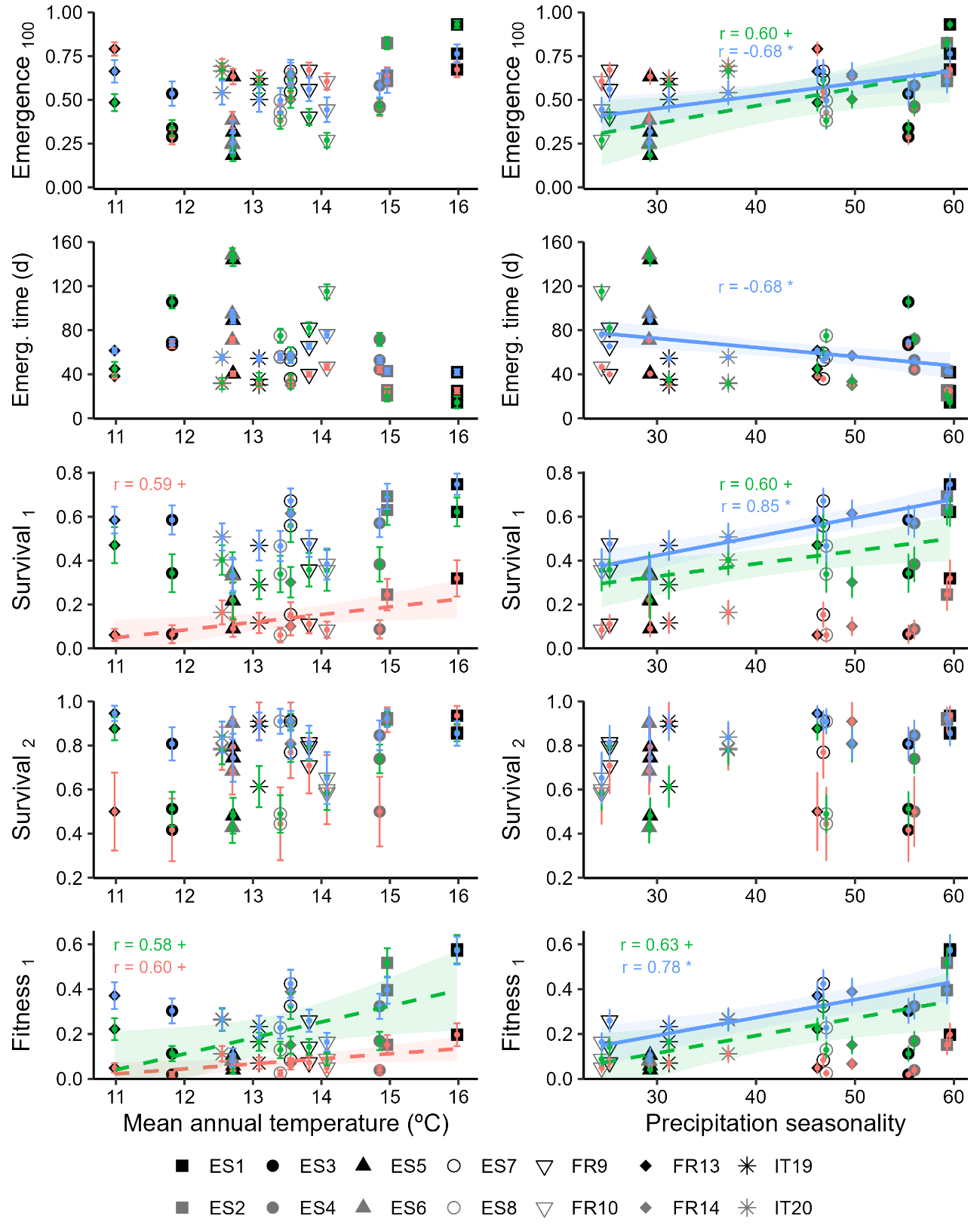


Figure S1. (Continued)


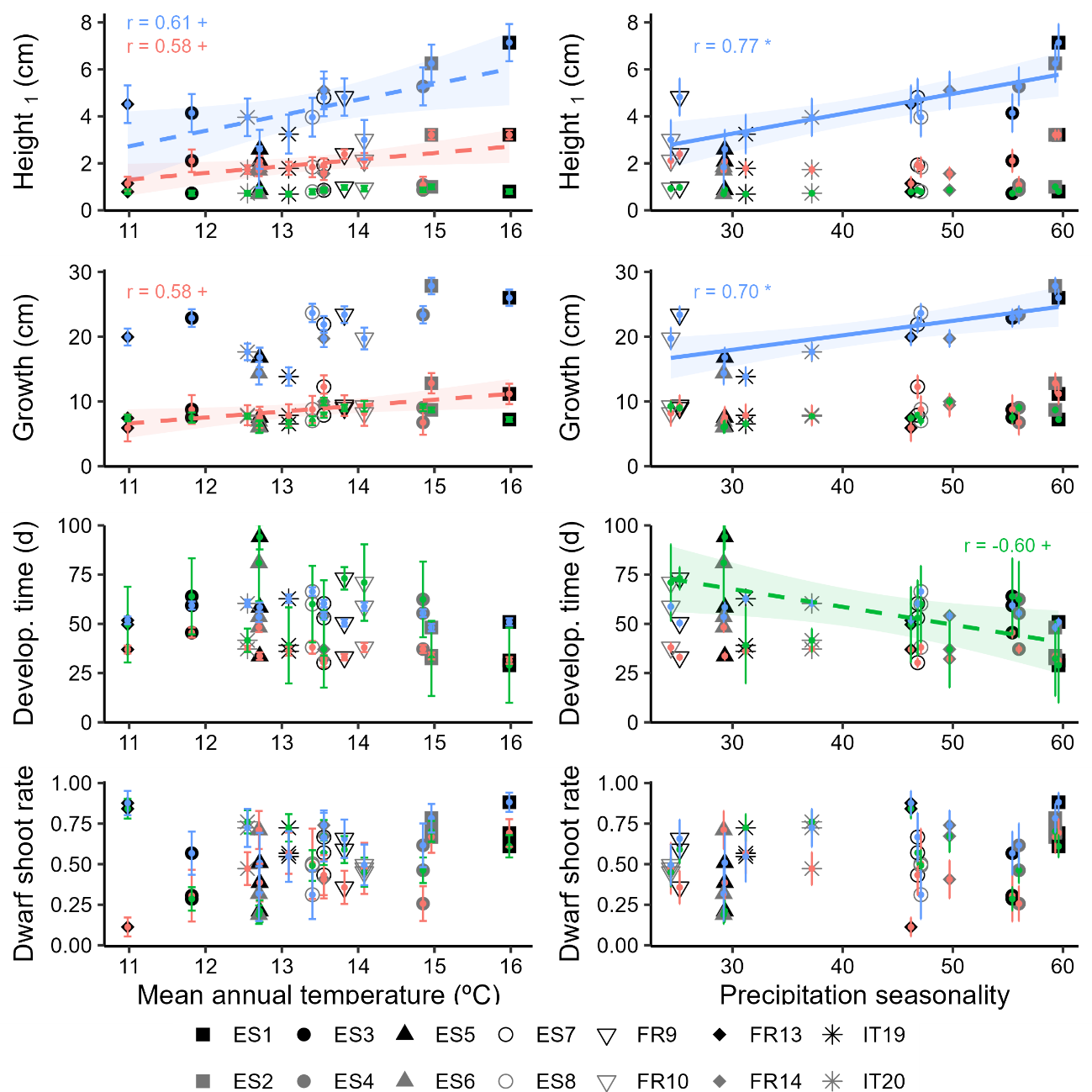

Supplement: mcae190_suppl_Supplementary_Tables_S1-S2_Figure_S1 [file mcae190_suppl_supplementary_tables_s1-s2_figure_s1.docx]
